# Biomedical markers and psychiatric morbidity of neurasthenia spectrum disorders in four outpatient clinics in India

**DOI:** 10.4103/0019-5545.42394

**Published:** 2008

**Authors:** V. P. Paralikar, M. M. Agashe, S. B. Sarmukaddam, H. N. Dabholkar, D. Gosoniu, M. G. Weiss

**Affiliations:** Maharashtra Institute of Mental Health, Sassoon Hospitals Campus, Pune - 411 001, India; 1 Regional Mental Hospital, Yerawada, Pune - 411 006, India; 2 Department of Public Health and Epidemiology, Swiss Tropical Institute, Basel, Switzerland

**Keywords:** Anthropometry, anxiety disorders, malnutrition, neurasthenia, somatoform disorders

## Abstract

**Context:**

Disorders of unexplained fatigue are researched globally and debated prominently concerning their biomedical and psychiatric comorbidity. Such studies are needed in India.

**Aims:**

To identify biomedical markers and psychiatric morbidity of disorders of severe unexplained fatigue or weakness with disability, designated neurasthenia spectrum disorders (NSDs). To compare biomedical markers of patients with controls. To study correlation between biomedical markers and psychiatric morbidity.

**Settings:**

Four specialty outpatient clinics of Psychiatry, Medicine, Dermatology, and Ayurved of an urban general hospital.

**Design:**

Case-control study for biomedical markers. Diagnostic interviews for assessment of psychiatric morbidity.

**Materials and Methods:**

Patients (N = 352) were recruited using screening criteria and Structured Clinical Interview for DSM-IV screening module. They were compared with controls (N = 38) for relevant biomedical markers. Psychiatric morbidity was assessed with SCID-I interviews, Hamilton scales, and Symptom Check List-90 (SCL-90). Correlations between a nutritional index and axis I morbidity were studied.

**Statistical Analyses:**

Frequencies and means of biomedical markers and psychiatric diagnoses were compared and associations assessed with regression analysis.

**Results:**

Corrected arm muscle area (CAMA) was significantly lower among patients (*P* < 0.001), but not anemia. Anxiety (73.0%) and somatoform (61.4%) disorders, especially nonspecific diagnoses, were more frequent than depressive disorders (55.4%). Generally, Hamilton and SCL scores were lowest in Ayurved clinic, and highest in Psychiatry clinic. Presence of Generalized Anxiety Disorder (GAD) and adjustment disorders correlated with low nutritional index.

**Conclusions:**

Malnutrition or de-conditioning that may explain weakness need to be considered in the management of NSDs in India, particularly with comorbid GAD or adjustment disorders. Weakness and anxiety, rather than fatigue and depression, are distinct features of Indian patients. SCL may be more useful than categorical diagnoses in NSDs. NSDs are an independent entity with nonspecific psychiatric comorbidity. Cross clinic differences among patients with similar complaints highlight need for idiographic studies.

## INTRODUCTION

Fatigue and weakness are common symptoms in medical practice. When the identifiable biomedical causes of fatigue are ruled out, unexplained fatigue and weakness are still very common.[1–4] These clinically significant conditions are classified with overlapping diagnostic categories,[5,6] such as neurasthenia, chronic fatigue syndrome (CFS) and fibromyalgia. Collectively, they may be regarded as neurasthenia spectrum disorders (NSDs),[7,8] which are a subset of functional somatic syndromes.[9] NSDs are heterogeneous conditions with poor diagnostic concordance among various categories.[8] NSDs have been studied extensively in Europe, Australia, and North America where their nomenclature, etiology, classification, and treatment have been debated. Biomedical investigation of NSDs in developed countries deal with the sophisticated immune and genetic mechanisms.[10–13] NSDs are often characterized by high psychiatric comorbidity, especially depression.[14–16] It is argued that neurasthenia is a subset of depression.[17] Whether or not psychiatric disorders are an integral feature of these conditions remains controversial.

NSDs are rarely diagnosed or researched in developing countries, though they are likely to become more significant with increasing urbanization.[18,19] There are no psychosomatic clinics in Indian general hospitals to study or treat the various psychosomatic illnesses including NSDs. A clinic-based study of NSDs documented its prevalence to be 5% raising questions about its biomedical and psychiatric correlates.[20] Clinical experience shows that patients with NSDs present for treatment in Indian urban general hospitals in a range of specialty clinics. Physicians in these clinics manage these patients according to their training specialty. This often leaves out comprehensive approach of biopsychosocial perspective resulting in restricted focus on comorbidity identified in respective clinics.

There are special challenges while dealing with NSDs in developing countries, where prevalence of infectious and nutritional deficiency disorders is high. Anemia was reported to be 44% in Indian men[21] and 50% in women.[22] Relationship between neuropsychiatric disorders and nutritional disorders is also well established. Considering the heterogeneity of origin of symptoms of fatigue or weakness, biomedical disorders remain a difficult yet important differential diagnosis of putative functional somatic syndromes in general, and of NSDs in particular. Scarcity of medical personnel and lack of availability or affordability of sophisticated investigations to gauge nutritional deficiencies is commonplace in developing countries. In a community prevalence survey among women in Goa, India, Patel *et al.*[28] considered nutritional and infectious disorders and psychopathology as risk factors of chronic fatigue.

The Indian *dhat* syndrome shares clinical features of neurasthenia, with symptoms attributed by patients to loss of semen.[23–27] The few relevant studies in India that do consider nosological status of *dhat* syndrome are anecdotal case reports.[24,27] Clinical experience shows that fatigue and weakness present in the contexts of different physical and psychiatric syndromes. Because they are poorly understood, doctors of different specialties offer different clinical explanations. Frustrated patients and their relatives entertain varied cultural meanings, as the condition becomes chronic. Also, inasmuch as conventional doctors do not provide satisfactory care, doctor-shopping and faith healing are common. Thus, these patients are seen in various clinics. Patients select a particular clinic or get referred from a previously attended clinic.

## AIMS

Biologic and psychiatric profiles of NSDs in Indian clinics have not been studied. This study was undertaken to clarify the biologic markers and psychiatric morbidity of patients with NSDs in four specialty out-patient clinics (Psychiatry, Medicine, Dermatology, and Ayurved) in an urban hospital in India. It aimed to compare biometric features of patients across clinics and with controls. We also aimed to compute an arbitrary nutritional index to examine the correlation between this index and the psychiatric morbidity.

## SUBJECTS AND METHODS

### Design

This study is a part of larger study of NSDs. In the context of routine hospital procedures, patients were enrolled based on spontaneously volunteered complaints of fatigue or weakness. For those patients who fulfilled the screening criteria, a physician was consulted. Trained research assistants screened the outpatients from the four study clinics. Those who screened positive and were willing and able to participate in this study, were recruited. For the study findings reported here, research interview included diagnostic interviews for SCID-I, HDARS, SCL-90 (enhanced) apart from laboratory investigations and biometric assessments.

### Setting and the four study clinics

This study was conducted in a large hospital with tertiary care facilities. It is attached to a Government Medical College and treats low-income patients through a State Government subsidy. The Ayurved clinic treats patients based on traditional Hindu system of medicine, which focuses on theories of humoral balance with reference to cultural physiologic concepts such as semen (*dhatu*), material substance (*prakriti*), and environmental conditions. Ayurved clinic is commonly valued by patients for its holistic approach, noninvasive methods, and fewer side effects. The Medicine clinic conforms to conventional biomedical clinical techniques. The Psychiatry clinic provides treatment for psychotic, mood, and other psychiatric disorders, and also follows standard international psychiatric guidelines. In addition to skin disorders, the Dermatology clinic also treats patients with sexually transmitted diseases, which are frequently associated with stigma.

### Instruments and assessments

#### (1) Screening criteria and the sample

To deal with the problem of overlapping international diagnostic categories[6] of different NSDs that are not used in Indian clinics routinely, we formulated the essential criteria of NSDs to include patients in this study. A positive screen for a NSD was based on the following criteria: (a) spontaneously reported complaints of fatigue or weakness among the presenting symptoms, (b) duration of symptoms for 6 months or more, (c) sufficient distress to motivate treatment seeking, (d) functional impairment with work or related activities, and (e) no biomedical basis for symptoms based on an evaluation by an internist physician considering clinical history, physical findings, standard laboratory studies, and clinically indicated tests.

Patients from the four study clinics between the ages of 17 and 65 who screened positive for NSD and were willing and able to complete the interview were recruited into the study after written informed consent. Those with overt psychotic disorders or substance use disorders were excluded from the study using the screening module of SCID.

Control patients were selected from the Psychiatry clinic, who met sampling criteria other than the core features of NSD. They were matched for age. Controls were selected only from the psychiatry clinic. They were nonpsychotic, nonsubstance dependant patients without the core features of NSDs. They were primarily afflicted by neurotic depression, anxiety, or somatoform disorders without fatigue or weakness. It has been argued that NSDs are only a manifestation of subclinical depression. Therefore, this set of controls gave us an opportunity to assess biomedical and anthropometric features of patients with the same psychiatric morbidity without NSDs.

#### (2) Biometric assessment

For study patients and controls, we assessed hemoglobin, body mass index (BMI), and corrected arm muscle area (CAMA) to assess nutritional factors. Height and weight were measured using standard methods for the computation of BMI. Triceps skin-fold thickness (using Harpenden's John and Bull callipers) and mid-arm circumference were measured leading to computation of CAMA.

#### (2) (a) Index of nutrition

An index of nutrition was computed arbitrarily by adding the values of hemoglobin, BMI, and CAMA for each patient represented by the quartile (range: 0-3) to which the patient belonged. The value of the index ranged from 0 to 9 for each patient with respect to all the three parameters. Subsequently, the mean index of nutrition for each clinic for each disorder was computed. For this, we considered patients for whom all three parameters were available. A higher value of the index indicated better nutritional status.

#### (3) Medical evaluation

Medical assessment to rule out infective and metabolic problems that might be responsible for core features of NSDs included estimation of hemoglobin by Sahli's method, total white blood cell counts (WBCs) by microscopic examination of the peripheral blood smear, fasting blood sugar level using Glucose Oxydase Peroxidase Kit, blood urea level estimation by Diacetyl Monoxime method, and routine and microscopic examinations of urine and stool samples. Other tests such as X-ray chest, electrocardiogram, thyroid tests, and HIV test were carried out wherever the internist physician found them necessary. The internist's opinion on clinical evaluation along with investigations decided whether patient's complaints were having a biomedical basis.

#### (4) Psychiatric assessment

For study patients only, an appointment for psychiatric assessment was made. Psychiatric diagnostic assessment used the research version of Structured Clinical Interview for DSM-IV for axis I (SCID-I).[29] Other assessments included the combined Hamilton Depression and Anxiety Rating Scales,[30] and Symptom Check-List 90[31] enhanced with a supplement to assess additional symptoms of fatigue or weakness. Instruments were translated into the local language (Marathi) using appropriate methods for validation, namely translation, back-translation, and consensus.

### Statistical analyses

Data for biometric and psychiatric assessments were entered in Epi Info 6.04d, using a data entry mask with range and logic checks. Double entry verified the accuracy of data entry. BMI, CAMA, hemoglobin, and other variables for biomedical evaluation were analyzed with a comparison of means across clinics, and between the controls and outpatient study groups. Age and sex adjusted means were computed for BMI, CAMA, and hemoglobin. The distribution of SCID-I diagnoses and mean scale scores of HDARS and SCL-90 assessments were compared across clinics, as well as subscale scores of the SCL-90 using appropriate tests of significance.

Comparison of the index of nutrition was done among patients with or without an axis I disorder. The differences between the means of patients not diagnosed with that disorder and those that were diagnosed were compared using *t*-test and parallel nonparametric test. Disorders for which frequency of available patients was < 10% were excluded from this analysis.

## RESULTS

### Sample characteristics

A total of 352 patients (with mean age 34.3 yrs, SD 10.34) were studied from the four clinics of Psychiatry, Medicine, Dermatology, and Ayurved. Most were literate, urban residents (67.0%), Hindu (76.1%), employed (53.1%), and earning an income of < Rs. 4000 per month (68.2%). The pooled sample had more women (63.8%), mainly housewives. Women constituted approximately half of the patient samples in Psychiatry (49.4%) and Dermatology (47.1%). Women were the majority in both the Medicine (87.8%) and Ayurved (67.4%) clinics. Patients in the Ayurved clinic were the oldest (mean age 37.7 years), and in Dermatology they were the youngest (mean age: 31.5 years).

Among 38 control patients, 20 males had a mean age of 35.3 years (SD: 8.35) and 18 females (mean age: 34.8, SD: 10.095). Among patients, males had a mean age of 31.4 years (SD: 10.58, *P* = 0.12) and females had a mean age of 36.0 years (SD: 9.85, *P* = 0.63).

### Identification of biomedical markers of NSDs

Patients recruited into the study had no signs of overt biomedical causes for their complaints of fatigue or weakness, as judged by an internist on clinical evaluation supported by laboratory tests. Total WBCs, fasting blood sugar and blood urea levels, and routine urinalysis and stool examinations of patients and controls were within normal limits.

### Nutritional assessments

The mean BMI of patients (21.48, *N* = 315) was similar to that of controls (21.92, *N* = 37) (*P* = 0.27, Mann-Whitney), and within the normal range for Asian population [Table 1]. Female patients had a higher mean BMI (22.15) than male patients (20.36).

**Table 1:** Sexwise distribution of hemoglobin levels (g/dl), BMI & CAMA (sq cm) for patients with NSDs and controls

Biometric parameter	Sex	Psychiatry		Medicine		Dermatology		Ayurved		*P*-value^a^	Total		Controls		*P*-value^b^
									
		*n*	Mean (SD)	*n*	Mean (SD)	*n*	Mean (SD)	*n*	Mean (SD)		*n*	Mean (SD)	*n*	Mean (SD)	
**Hb (g/dl)**	Male	38	12.75	12	11.83	41	12.54	26	13.53	**0.0004**	117	12.76	20	11.75	**0.0019**
			*1.15*		*0.58*		*1.20*		*1.49*			*1.29*		*1.13*	
	Female	39	10.5	82	10.45	37	10.76	51	11.51	**0.0001**	209	10.77	14	10.71	0.9811
			*1.18*		*1.06*		*0.90*		*1.50*			*1.25*		*1.55*	
	Both	77	11.61	94	10.62	78	11.69	77	12.2	**<0.0001**	326	11.49	34	11.32	0.8244
			*1.62*		*1.11*		*1.39*		*1.77*			*1.58*		*1.40*	
**Sex-adjusted Mean Hb BMI**			11.31		10.94		11.4		12.23			11.53		11.11	
	Male	40	21.18	12	19.55	43	20.01	23	19.99	0.5997	118	20.36	20	21.06	0.2273
			*3.84*		*1.9*		*2.55*		*3.74*			*3.25*		*2.55*	
	Female	40	23.03	80	21.7	36	23.37	41	21.12	0.1724	197	22.15	17	22.93	0.3572
			*4.58*		*4.68*		*6.19*		*4.18*			*4.92*		*4.18*	
	Both	80	22.1	92	21.42	79	21.54	64	20.72	0.3111	315	21.48	37	21.92	0.2688
			*4.3*		*4.47*		*4.85*		*4.03*			*4.45*		*3.48*	
**Sex-adjusted Mean BMI**			22.34		20.89		22.14		20.7			21.45		22.20
**CAMA (sq cm)**	Male	40	33.22	12	29.98	40	28.61	23	37.02	0.3846	115	32.04	20	39.03	**0.0301**
			*11.97*		*14.77*		*13.91*		*12.60*			*13.30*		*10.54*	
	Female	39	28.47	79	18.61	35	27.37	40	29.84	**<0.0001**	193	24.52	17	39.94	**0.0005**
			*8.67*		*16.14*		*11.62*		*11.35*			*13.99*		*13.90*	
	Both	79	30.86	91	20.11	75	28.04	63	32.46	**<0.0001**	308	27.33	37	39.45	**<0.0001**
			*10.68*		*16.35*		*12.82*		*12.23*			*14.19*		*12.03*	
**Sex-adjusted Mean CAMA**			30.34		23.08		27.86		32.66			27.46		39.58	

a Kruskall-Wallis test.

b Mann-Whitney test, SD values expressed in *italics*, Significant P values expressed in **bold**

Mean CAMA for patients (27.33 sq cm, *N* = 308) was significantly lower than controls (39.45 sq cm, *N* = 37, *P* < 0.001, Mann-Whitney). The mean CAMA for female cases (24.52 sq cm) was significantly lower than male cases (32.04 sq cm) (*P* < 0.001), but there was little difference between sexes among controls (*P* = 0.94, Mann-Whitney). Across clinics, Medicine patients had the lowest mean CAMA scores and Ayurved patients had the highest mean CAMA scores (*P* < 0.001) [Table 1].

Mean hemoglobin value of patients (11.49 g/dl, *N* = 326) was similar to that of controls (11.32 g/dl, *N* = 34, *P* = 0.82). Medicine clinic patients had significantly lower mean hemoglobin values (10.62 g/dl) than other clinics [Table 1].

The overall comparison of means between study patients and controls showed that there was no difference regarding hemoglobin and BMI. CAMA was significantly lower among females from each clinic, and also among males from Dermatology clinic [Table 2]. Males in the Dermatology clinic had a lower BMI, and both males and females from Medicine clinic had lower BMI, though the difference was not significant. BMI was lower among younger age groups. Males had lower BMI than females.

**Table 2:** Sexwise distribution of mean difference in Hemoglobin levels (gm%), BMI & CAMA (sq cm) in patients with NSDs and controls

Variable	Sex	Psychiatry		Medicine		Dermatology		Ayurved	
					
		Mean difference^a^	*P* value^b^	Mean difference^a^	*P* value^b^	Mean difference^a^	*P* value^b^	Mean difference^a^	*P* value^b^
Hb	Male	1.000	**0.003**	0.080	0.981	0.790	**0.022**	1.780	**<0.001**
	Female	-0.214	0.888	-0.264	0.756	0.046	1.000	0.796	0.071
	Both	0.290	0.570	-0.700	0.059	0.370	0.390	0.880	**0.008**
BMI	Male	0.120	0.999	-1.510	0.477	-1.050	0.535	-1.070	0.618
	Female	0.100	1.000	-1.230	0.646	0.440	0.988	-1.810	0.413
	Both	0.180	0.995	-0.500	0.925	-0.380	0.975	-1.200	0.429
CAMA	Male	-5.810	0.309	-9.050	0.179	-10.420	0.014	-2.010	0.976
	Female	-11.470	**0.010**	-21.330	**<0.001**	-12.570	0.005	-10.100	**0.026**
	Both	-8.590	**0.005**	-19.340	**<0.001**	-11.410	**<0.001**	-6.990	**0.026**

a Mean difference indicates subtraction between values for cases and controls; negative values indicate cases having smaller values than controls.

b *P*-value was computed by Dunnett test for multiple comparisons between cases and controls for individual clinics. Significant *P* values expressed in **bold**.

A multivariate regression was performed to study the association between biomedical markers and psychiatric disorders (with female sex, 31-40 year age-group, and controls as the baseline). After controlling for confounding, the multivariate analysis indirectly indicated that patients from the Ayurved clinic had a lower BMI than the controls (*P* = 0.02).

The differences regarding hemoglobin in the crude analysis between patients and controls remained insignificant after adjustment for sex and clinic (with female sex and controls as baseline). The differences in CAMA between the sexes did not remain after adjustment for sex and clinic. Multivariate analysis with sex, age, and clinic confirms findings of the univariate analysis.

Regarding the index of nutrition [Table 3], only those patients from each clinic were considered for whom all three parameters (hemoglobin, BMI, and CAMA) were available. They were 75 in psychiatry, 90 in medicine, 73 in dermatology, and 57 in Ayurved (*N* = 295). Negative value of the mean difference indicates better nutrition for diagnosed patients in that clinic. For example, being diagnosed with social phobia in psychiatry, and minor depressive disorder or undifferentiated somatoform disorder in medicine clinic were associated with better nutrition than when these disorders were not diagnosed, indicating an absence of nutritional basis with these diagnoses. On the other hand, positive mean difference between those without and with a disorder indicates lower mean index of nutrition for diagnosed patients. For example, mean difference of 2.16 and 0.64 in Ayurved clinic and among total patients, respectively, indicates that generalized anxiety disorder was significantly associated with undernutrition. For patients with core features of NSDs, diagnoses of past major depressive episode, anxiety disorder not otherwise specified (NOS), and undifferentiated somatoform disorder indicated higher nutritional level, but being diagnosed with adjustment disorder was associated with poor nutritional status.

**Table 3:** Nutritional status of NSD patients: Comparison of mean index of nutrition of patients with and without psychiatric disorders

Variable	Psychiatry (*n* = 75)		Medicine (*n* = 90)		Dermatology (*n* = 73)		Ayurved (*n* = 57)		TOTAL (*N* = 295)	
					
	Mean diff	*P* value	Mean diff	*P* value	Mean diff	*P* value	Mean diff	*P* value	Mean diff	*P* value
Current major depressive episode	0.05	0.9137	0.75	0.2171	0.61	0.3593	1.47	0.1390	0.36	0.2712
CMDE with melancholic features	-0.01	0.9909	0.60	0.5250	0.70	0.5616	1.00	0.6698	0.30	0.5705
Past major depressive episode	-0.64	0.2245	-0.79	0.4393	-1.55	0.1177	-0.02	0.9844	-1.07	**0.0097**
Dysthymic disorder	-0.50	0.4062	0.06	0.9541	-1.08	0.2153	2.02	0.3883	-0.64	0.3269
Minor depressive disorder	-3.15	0.1116	-1.53	**0.0129**	-1.61	0.4943	0.59	0.4691	-0.40	0.3970
Panic disorder	-0.34	0.6011	0.06	0.9541	1.47	0.2853	-	-	-0.04	0.9497
Social phobia	-1.39	**0.0021**	-0.12	0.8990	-0.65	0.4838	2.09	0.1253	-0.59	0.2167
Specific phobia	-0.75	0.2837	-0.66	0.3529	0.87	0.4219	1.54	0.3571	-0.03	0.9464
Generalized anxiety disorder	-0.24	0.7232	0.07	0.8925	0.09	0.9155	2.16	**0.0122**	0.64	0.0769
Anxiety disorder NOS	-0.40	0.5901	-0.56	0.4336	-0.73	0.2441	-0.88	0.2182	-0.79	**0.0252**
Pain disorder	-0.22	0.7003	0.69	0.1641	-0.42	0.6159	0.11	0.8954	0.47	0.1511
Undifferentiated somatoform disorder	-0.29	0.5955	-1.54	**0.0053**	-1.02	0.0612	-0.11	0.8618	-0.97	**0.0007**
Adjustment disorder	-0.12	0.8721	0.85	0.1000	0.79	0.2835	-0.39	0.6342	0.75	**0.0302**

*Notes*: Negative value indicates higher mean index of nutrition for diagnosed patients Positive value indicates lower mean index of nutrition for diagnosed patients *P* value is based on *t*-test comparing the difference between means of indices of patients with and without a disorder. Significant *P* values expressed in **bold**.

### Psychiatric assessments

Table 4 summarizes psychiatric disorders based on the SCID-I interviews, and illustrates that patients mostly suffered from common mental disorders. Comorbidity was found in nearly all patients, and only 6.5% had no diagnosis. Anxiety disorders were the most frequent diagnoses, and among them, anxiety disorder NOS was the most commonly diagnosed category, followed by somatoform disorders, and depression.

**Table 4:** Psychiatric diagnoses of patients with CS-FAW disorders (%)

Diagnosis	Psychiatry (*n* = 83)	Medicine (*n* = 98)	Dermatology (*n* = 85)	Ayurved (*n* = 86)	Total (*N* = 352)	*P* value (Chi sq)
Depressive disorders	86.7	48.0	47.1	41.9	55.4	<0.001
Current major depression	65.1	26.5	31.8	20.9	35.5	**<0.001**
Past major depression	26.5	6.1	9.4	9.3	12.5	**<0.001**
Dysthymic disorder	16.9	6.1	11.8	4.7	9.7	**0.026**
Minor depressive disorder	1.2	18.4	3.5	14.0	9.7	**<0.001**
Anxiety disorders	81.9	77.6	75.3	57.0	73.0	0.001
Panic disorder	13.3	7.1	4.7	0.0	6.3	**0.004**
Panic disorder with agoraphobia	6.0	4.1	1.2	0.0	2.8	-
Agoraphobia without panic	4.8	7.1	4.7	3.5	5.1	-
Obsessive compulsive disorder	2.4	0.0	2.4	0.0	1.1	-
Social phobia	12.0	6.1	8.2	5.8	8.0	0.408
Specific phobia	10.8	13.3	7.1	3.5	8.8	0.102
Post-traumatic stress disorder	4.8	2.0	1.2	0.0	2.0	-
Generalized anxiety disorder	12.0	25.5	11.8	14.0	16.2	**0.031**
Anxiety disorder NOS	10.8	11.2	28.2	26.7	19.0	**0.001**
Mixed anxiety depression	4.8	1.0	5.9	3.5	3.7	0.331
Somatoform disorders	51.8	62.2	64.7	66.3	61.4	0.212
Somatization disorder	4.8	3.1	1.2	0.0	2.3	-
Pain disorder	20.5	29.6	12.9	12.8	19.3	**0.010**
Undifferentiated somatoform disordera	21.7	23.5	45.9	51.2	35.2	**<0.001**
Hypochondriasis	3.6	6.1	4.7	1.2	4.0	-
Body dysmorphic disorder	1.2	0.0	0.0	1.2	0.6	-
Adjustment disorders	9.6	25.5	15.3	15.1	16.8	0.131
No diagnosis	4.8	7.1	5.9	8.1	6.5	0.830

^a^Diagnosis based on symptoms other than fatigue and weakness. - : Not calculable. Significant *P* values expressed in **bold**.

In the cross-clinic comparisons, the Psychiatry clinic had the highest rates of enduring psychiatric disorders (85.5%), attributable mainly to highest rates of all forms of depression, except minor depressive disorder, which was high in the Medicine and Ayurved clinics. Based on exclusions for depressive and anxiety disorders, Psychiatry patients had lower rates of somatoform disorders and adjustment disorders. Rates of current and past major depressive episodes and of dysthymic disorder were relatively high in dermatology clinic patients. Because of low rates of panic disorders and phobias, overall anxiety was least frequent in the Ayurved clinic, though rates of anxiety disorder NOS were significantly higher for both Dermatology and Ayurved than the other clinics. Undifferentiated somatoform disorder, based on symptoms apart from fatigue and weakness, was also significantly more frequent in Ayurved and Dermatology. Pain disorder was the highest in the Medicine clinic.

**Figure 1:**
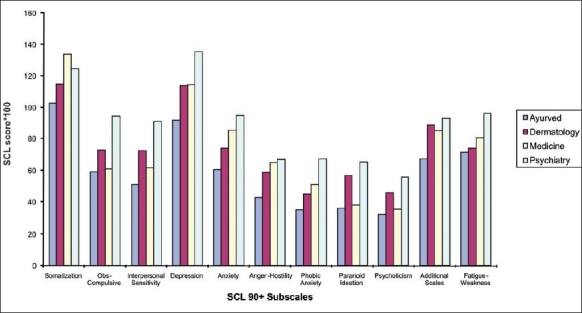
Distribution of mean SCL 90+ subscale scores

For dimensional scale scores, Psychiatry patients had highest mean values of both Hamilton depression (HDRS) and anxiety (HARS) rating scales. The respective mean scores for HDRS were 17.5 (7.6 SD) in Psychiatry, 16.5 (7.3 SD) in Medicine, 14.5 (8.2 SD) in Dermatology, and 12.4 (8.2 SD) in Ayurved. Mean HARS scores were 14.1 (6.9 SD) in Psychiatry, 13.7 (6.5 SD) in Medicine, 10.8 (6.2 SD) in Dermatology, and 10.7 (6.4 SD) in Ayurved. Differences for both were highly significant (*P* < 0.001, Kruskal Wallis) across clinics [Figure 1], indicating the magnitude of SCL-90+ subscale scores in the four clinics, shows that for all subscales except somatization, patients in Psychiatry had the highest mean values. All subscale scores were the lowest for Ayurved clinic patients.

## DISCUSSION

Though fatigue and weakness are universal as symptoms, various disorders of biomedically unexplained fatigue and weakness, such as CFS and neurasthenia have poor diagnostic concordance.[8] These categories are rarely used in practice in Indian setting, and rarely researched, perhaps because these categories fail to provide adequate guidelines for clinical management. Each of these categories has additional criteria beyond the essential common features of a fatiguing disorder, viz., severe fatigue causing disability without obvious biomedical basis. These additional criteria make these categories useful in the setting where the condition was formulated, but these categories lose their sensitivity in other cultures. Cross-cultural differences in disorders of fatigue and weakness are important. Broad and inclusive criteria with focus on clinical significance alone were used in this study to screen patients from four different clinics to circumvent the problem of culture bound syndromes. The four clinics represent distinct medical, psychiatric, and cultural orientations to the experience of illness, its explanations, and preferred help. Present study describes the salient biomedical and psychiatric differences among patients attending four different clinics with different cultural orientations toward health and disease. Considering the range of variation with respect to clinical features, biomedical status, and psychopathology, experience in the study supports the validity of applying the formulation of NSD to this sample and others.

It is notable that patients could be identified with a common case definition in each of the four clinics. Study patients meeting inclusion criteria for core features of NSDs were shown to have differences in biomedical and psychopathologic markers across the four clinics.

### Endemic anemia

Study patients were not more anemic than the controls, a finding similar to the one reported by Patel *et al*.[28] But Indian patients are more anemic as compared to their counterparts in other parts of the world. Thus, “weakness,” which is not emphasized in Western definitions of these disorders, may be partly explained by anemia of Indian patients.

Nearly all male controls (95%) and 74% of male patients had < 14 g/dl of hemoglobin, and almost 80% of both female patients and controls had it < 12 g/dl. These international standards are rarely followed in clinical practice in India, where treatment is started only when hemoglobin is < 10.5 g/dl in the local setting. Patel and colleagues in their community study of Indian women also take the cut-off as 11 g/dl for non-pregnant women.[28] Other population studies have shown that 50% women and 44% men have anemia in India.[21,22]

The study sample showed comparable values of hemoglobin with controls. It is to the credit of clinicians in busy outpatient clinics with unsatisfactory doctor patient ratio and insufficient time and resources for investigations that there was no significant difference between mean values for hemoglobin and BMI of patients and controls. This indicated that they had identified patients with no biomedical basis correctly based on clinical evaluation. It is noteworthy that BMI and CAMA are not done routinely in the clinics of the hospital where study was conducted. The differences in the values of CAMA between patients and controls show a deficiency in the assessment of patients with NSDs regarding biomedical basis of their complaints. Although it is not the most sophisticated investigation for deficiency of muscle mass (sarcopenia), CAMA is a simple, inexpensive, and proven indicator of nutritional status that should be used to screen and identify patients with nutritional basis for their NSDs in public hospitals facilitating further work-up.

### Distinctive CAMA

Based on the inclusion criteria, there should have been few differences in biomedical markers between cases and controls. This was true for anemia, but not for CAMA. CAMA was significantly higher in controls. Mean values of CAMA in urban slum dwellers more than 50 years of age from Mumbai were 35.3 sq cm for men and 27.8 sq cm for women.[32] Our study patients’ values of 32.04 sq cm for men and 24.52 sq cm for women are close to these Indian norms. Having sufficiently ruled out medical causes, undernutrition, or lack of activity (or both) could yield low values on anthropometric measurements. Sarcopenia detected in study patients may be partly explained by more female patients and the younger age of male patients from Dermatology (as is endorsed by findings from multivariate analyses mentioned above), or from their impaired work capacity, which was an inclusion criterion. Less activity is also known to be a cause of lower CAMA.[33–35] Their self-perceived health also was poor.

Deconditioning is an important perpetuating factor for complaints of fatigue, and also important in poor compliance with therapeutic suggestions regarding activity.

Sarcopenia may be another explanation for the complaints of weakness in our study patients. It is rarely a focus of inquiry in Western definitions of disorders of fatigue. Further studies on sarcopenia will be important for developing effective treatment protocols.

### Gender issues in NSDs

Undernutrition of women is not only a biologic issue, but also a problem that requires attention to sociocultural determinants arising from gender-based vulnerability of women. Their higher prevalence in women is comparable with findings from other studies.[3]

### NSDs as distinct from depression: corroboration from controls

Controls were found to be dissimilar from patients regarding CAMA. It strengthens the argument that NSDs are not just unremitted or subclinical depression or other psychiatric morbidity, but a category in their own right with distinctive anthropometric features. These findings need to be studied in larger samples, as it was not possible to recruit more controls in a setting that largely treats severe mental morbidity. Also, more sophisticated investigations of lower CAMA in patients with NSDs are necessary to examine the correlation between sarcopenia and prominence of weakness in clinical presentation.

### Frequency and multiplicity of psychiatric diagnoses

Preponderance of anxiety and weakness in this study contrasts with that of fatigue and depression in Western studies. It is a principal cross-cultural difference for disorders that comprise the spectrum of NSDs, such as CFS and neurasthenia. Patients with clinically significant psychopathology, who did not meet criteria for enduring DSM-IV disorders were diagnosed with nonspecific subtypes of somatoform, anxiety, or depressive disorders, or with adjustment disorders. Other studies have shown that for patients with medically unexplained symptoms[15] or CFS,[4,14] the frequency of depression is greater, followed by anxiety and somatoform disorders. The majority of patients in this study received diagnoses of anxiety disorders or somatoform disorders, but few received depressive diagnoses, distinctive to urban Indian patients. Distinct profiles of psychiatric diagnoses across clinics indicate a preponderance of nonspecific anxiety and somatoform disorders in Dermatology and Ayurved clinics. For example, undifferentiated somatoform disorder (35.2%) and anxiety disorder NOS (19.0%) are more frequent in the Dermatology and Ayurved clinics. Prevalence of unexplained fatigue or weakness was also shown to be more in these two clinics than the Psychiatry and Medicine clinics, indicating important differences in the way these symptoms are perceived and presented in these clinics. This warrants a detailed assessment of NSDs in these clinics. It is possible that the theme of psychiatric suffering in these clinics is different from the other clinics, and based more on cultural tenets. Quality of experience in depression needs to be explored, as stressful life events including victimization are known to be associated with NSDs. Adjustment disorders are related to real-life stresses that are reported most by medicine clinic patients, largely women. Financial stresses are common perhaps linked with nutritional problems.

These findings show that NSDs are not a subset of psychiatric morbidity, but rather nonspecific psychiatric diagnoses are an associated feature of NSDs. This argument is in accordance with that of Henningsen[36] indicating the independent nature of functional somatic syndromes. High incidence of anxiety, minor depression, and pain disorder in the Medicine clinic highlights the need to manage untreated emotional problems of medical outpatients. Striking similarity between findings and implications of this study with that of Thieme[37] examining fibromyalgia is eloquent criticism on cross-cultural comparison of NSDs. They also concluded that fibromyalgia is a heterogeneous disorder requiring attention to physical as well as emotional problems.

The analysis of mean index of nutrition in each clinic and pooled sample shows that minor depressive disorder, social phobia, anxiety disorder NOS, and undifferentiated somatoform disorder generally indicated no major nutritional problems for the patients. Generalized anxiety disorder and adjustment disorder on the other hand indicated poorer nutritional status implying the need for investigation and more support for these patients.

Significant differences in the subcategories and magnitudes of depression and anxiety across clinics may indicate why these patients with similar symptoms seek help from different providers. For example, those with high-somatic anxiety may be attending medicine clinic, while those with high depression attend psychiatry clinic. Cultural physiologic beliefs may generate nonspecific psychiatric diagnoses, which are more frequent among patients in Dermatology and Ayurved clinics. Overall, the categorical diagnoses are of limited utility in managing these patients. SCL provides dimensional measurement, which is congruent with their degree of distress as indicated by Hamilton scores across clinics. Dimensional measurement of psychopathology may be a useful and complementary approach in professional assessments that will help patients understand, acknowledge, and monitor their own symptoms. Patel *et al.*[28] also used nonpsychotic psychiatric morbidity scores as indicators of psychopathology; this approach is analogous to the use of SCL 90. Somatization was the strongest predictor of both new and chronic fatigue with unknown cause as reported in the Epidemiological Catchment Area (ECA) study.[38] The present study of patients with NSDs corroborates these findings across cultures. Distinctive biomedical markers and psychopathology in the four clinics further highlighted the value of both cross-cultural and intra-cultural study of this common clinical problem from a biopsychosocial perspective. Idiographic formulation[39] is the assessment complementary to the professional assessments, such as SCID, Hamilton, and SCL-90. Such formulations could be obtained by cultural epidemiological methods for qualitative and quantitative measurements. Such research will contribute to the management of this vexing yet common clinical problem.
